# Phenolic Profile of *Croton urucurana* Baill. Leaves, Stems and Bark: Pairwise Influence of Drying Temperature and Extraction Solvent

**DOI:** 10.3390/molecules25092032

**Published:** 2020-04-27

**Authors:** Jáliston Júlio Lopes Alves, Maria Inês Dias, João C. M. Barreira, Lillian Barros, Osvaldo Resende, Ana Carolina Ribeiro Aguiar, Isabel C. F. R. Ferreira

**Affiliations:** 1 Centro de Investigação de Montanha (CIMO), Instituto Politécnico de Bragança, Campus de Santa Apolónia, 5300–253 Bragança, Portugal; jaliston.alves@ifgoiano.edu.br (J.J.L.A.); maria.ines@ipb.pt (M.I.D.); iferreira@ipb.pt (I.C.F.R.F.); 2 Laboratório de Pós-colheita de Produtos Vegetais, Instituto Federal Goiano—Campus Rio Verde, 75.901–970 Rio Verde, Goiás, Brazil; osvresende@yahoo.com.br (O.R.); ana.carolina@ifgoiano.edu.br (A.C.R.A.)

**Keywords:** *Croton urucurana*, extraction solvent, drying temperature, phenolic profile

## Abstract

Cerrado biome represents an area with great biodiversity. Some of its plants have significant ethnopharmacological uses, with specific purposes. *Croton urucurana* Baill., for instance, was previously acknowledged for its anti-hemorrhagic, anti-inflammatory, antiseptic, healing, and potentially antifungal and entomopathogenic actions. Nevertheless, the compounds supporting these empirical applications are still unknown. Accordingly, this work was designed to achieve a complete characterization of the phenolic profile of different botanical tissues obtained from *C. urucurana*, and also to verify how different operational conditions (different drying temperatures and extraction conditions) affect that profile. All samples were further characterized by HPLC–DAD–ESI/MSn, and results were compared by advanced chemometric tools. In general, the drying temperatures that maximize the extraction yield of specific individual phenolic compounds were established. Likewise, it was possible to verify that samples extracted with the hydroethanolic solution allowed higher phenolic yields, either in individual compounds (except (epi)catechin-di-*O*-gallate) or total phenolics. The identification of the best operational conditions and phenolic profiles associated with each *C. urucurana* botanical part contributes to enabling their use in food or pharmaceutical-related applications.

## 1. Introduction

Man has been using plant species for a variety of purposes (mainly related to food and medicinal applications) since ancient times [1,2,3]. The ethnopharmacological use of plants is essentially based on their effectiveness in alleviating different symptoms or their potential capacity to treat or prevent specific diseases [3,4]. These applications are especially noticeable in areas with high biodiversity, as in the case of Cerrado region. In fact, the Cerrado biome has one of the largest numbers of flora in the world, estimated at approximately seven thousand species, which is highly reflected in the cultural framework of the regional populations [2]. Despite this advantageous biodiversity, the exploitation of medicinal plants can be seriously limited by ineffective extractions, by the lack of thorough information about the bioactive compounds present in plants, or simply due to unsuitable drying and storage methods [1].

Accordingly, a great deal of effort has been dedicated by the scientific community to identify the compounds providing the physiologic effects pursued in ethnopharmacological uses, improve their extraction conditions and purity degrees, and ultimately to contribute to the preservation of important natural resources [3,4].

One of the tree species with wider ethnopharmacological uses among Cerrado species is represented by *C. urucurana* Baill, which is popularly known as “*sangra d′água*”. *C. urucurana* has been used for its anti-hemorrhagic, anti-inflammatory, antiseptic, healing, and potentially antifungal and entomopathogenic properties [5,6]; it is characterized as being a tall tree (10–12 m), with dark red sap, found in regions of tropical climate, such as Paraguay, Uruguay, Argentina, and Brazil. It is exclusively (or predominantly) found among riparian vegetation (interface between land and a river or a stream) [7]. As commonly verified among plant species used for phytotherapeutic purposes, the botanical tissues with highest potential are the leaves, branches, and bark, owing to their larger amounts of bioactive compounds [8]. The red-toned sap of *C. urucurana*, for instance, exhibits a wide variety of metabolites produced in the chiquimic acid and acetate pathways, such those as exemplified by different diterpenoids. In fact, the *Croton* genus is one of the richest sources of clerodane (especially in the bark) and clerodane diterpenes (e.g., methyl-3-oxo-12-epibarbascoate, sonderianin, and 15,16-epoxyclerodan-3,13(16),14(15)-trien-2-one). Other important compounds include acetylaleuritolic acid, stigmasterol, campesterol, β-sitosterol, β-sitosterol-3-*O*-glucoside, catechin, and gallocatechin [6,9].

In any case, the quality of these compounds is highly influenced by the employment of effective processing conditions (immediately after harvest) with the capacity to preserve those bioactive compounds and increase their possible use in phytotherapeutic products [10]. Actually, optimizing drying and storage processes, aiming at excellent post-harvest quality, is essential to maintain all the chemical properties found in fresh plants. In addition, and considering a practical point of view, industrial facilities are not structurally prepared to use fresh plants at large scale [11,12].

However, according to our knowledge, there is still no study on the influence of drying temperature on the phenolic composition of *C. urucurana*. Bearing this in mind, the individual phenolic profiles of *C. urucurana* samples (leaves, branches, and barks) dried at different temperatures (40, 50, 60, and 70 °C) and further extracted using different solvents (water and water:ethanol 80:20 *v*/*v*) were thoroughly compared to define the most suitable processing conditions (highest yield of phenolic compounds).

## 2. Results and Discussion

### 2.1. Phenolic Profiles of Leaves, Stems, and Bark of C. urucurana

The phenolic compounds characterization was performed on the hydroethanolic extracts and infusions preparations of leaves, bark, and stems of *C. urucurana* Baill. The choice of the extraction solvents/preparations was based on the traditional use of this plant, in the case of decoction extract, and in the fact that different percentages of polar and nonpolar solvents can maximize the extraction of phenolic compounds [13]. In order to keep a reasonable complexity of variables, only one water:ethanol solvent concentration was studied. The chromatographic characteristics of the individual compounds are summarized in Table 1. Overall, twenty-six compounds were identified, with a marked presence of sixteen flavonols (quercetin, myricetin, kaempferol, isorhamnetin, and syringetin) glycosyl derivatives, followed by six flavan-3-ols ((epi)catechin derivatives), three *C*-linked flavone glycosyl derivatives (apigenin), and one phenolic acid (gallic acid). The presence of *C*- linked flavonoid derivatives as the main compounds found in the *Croton* species is in accordance with [14]. However, as far as the authors know this is first report describing the individual phenolic profile of *C. urucurana* Baill. Peaks 1, 6, 11, 15, 18, 19, 20, 21, and 23 were identified as gallic acid, catechin, myricetin-3-*O*-rutinoside, apigenin-8-*C*-glucoside, quercetin-3-*O*-rutinosde, apigenin-6-*C*-glucoside, quercetin-3-*O*-glucoside, kaempferol-3-*O*-rutinoside, and isorhamnetin-3-*O*-rutinoside, respectively, by comparison of their chromatographic characteristics with available standard compounds. Catechin has been previously identified in the ethanolic extracts of *C. urucurana* bark [15].

As mentioned above, flavonoids were the main compounds identified, especially quercetin glycosyl derivatives, thus peaks 9 ([M − H]^−^ at *m*/*z* 625), 12 ([M − H]^−^ at *m*/*z* 741), 13, 14, and 16 ([M − H]^−^ at *m*/*z* 771), and 17 ([M − H]^−^ at *m*/*z* 609), were tentatively identified as derivatives of this flavonol, revealing a λ_max_ around 350 nm and releasing a MS^2^ (second stage of mass spectrometry)fragment at *m*/*z* 301, respectively corresponding to the losses of two hexosyl moieties [M − H − 162 − 162]^−^, a deoxyhexosyl and hexosyl moiety [M − H − 146 − 162]^−^, a hexosyl and rutinosyl moiety [M − H − 162 − 308]^−^, and a pentosyl and rutinosyl moiety [M − H − 132 − 308]^−^, being tentatively assigned as quercetin-*O*-dihexoside (peak 9), quercetin-*O*-pentosyl-rutinoside (peak 12), quercetin-*O*-hexosyl-rutinoside I/II/III (peaks 13, 14, and 16), and quercetin-*O*-deoxyhexosyl-hexoside (peak 17). Peak 10 presented the same pseudomolecular ion [M − H]^−^ at *m*/*z* 625 as peak 11 (myricetin-3-*O*-rutinoside), releasing a unique MS^2^ fragment at *m*/*z* 317 (myricetin aglycone) indicating the simultaneous loss of a deoxyhexosyl and a hexosyl groups [M − H − 146 − 162]^−^, tentatively identified as myricetin-*O*-deoxyhexosyl-hexoside. The same occurred with peak 21 that presented a λ_max_ at 348, a pseudomolecular ion [M − H]^−^ at *m*/*z* 593, and a unique MS^2^ fragment at *m/z* 285 (kaempferol aglycone), tentatively identified as kaempferol-*O*-deoxyhexoxyl-hexoside. Similarly, peak 24 ([M − H]^−^ at *m*/*z* 623) was tentatively identified as isorhamnetin-*O*-deoxyhexoside-hexoside. The last *O*-glycosylated flavonoid was tentatively identified as syringetin-*O*-rutinoside ([M − H]^−^ at *m*/*z* 653), releasing a MS^2^ fragment at *m*/*z* 345 (syringetin aglycone), which corresponded to the loss of a rutinosyl moiety [M − H − 308]^−^.

Peak 8 ([M − H]^−^ at *m*/*z* 593) released MS^2^ fragments that corresponded to the loss of 120 and 90 u characteristic of *C*-hexosyl flavones [16], thus being tentatively identified as apigenin-6,8-*C*-dihexoside.

Peaks 4, 5, and 7 were identified as proanthocyanidins (PAC) based on their pseudomolecular analysis and MS^2^ fragmentation patterns. Hereupon, peaks 4 and 5 were identified as (epi)catechin dimers isomer I and II, respectively, and peak 7 as (epi)catechin tetramer, presenting a pseudomolecular ion [M − H]^−^ at *m*/*z* 577 and 1153, respectively and MS^2^ fragmentation patterns coherent with B-type (epi)catechin dimers and tetramers, as previously described by Dias et al. [17]. Peaks 2 ([M − H]^−^ at *m*/*z* 305) and 3 ([M − H]^−^ at *m*/*z* 593) were also assigned as flavan-3-ol, particularly as (epi)gallocatechin and (epi)gallocatechin–(epi)catechin, respectively, based on its absorption, mass spectra, and bibliographic reference [18].

### 2.2. Effect of Drying Temperature and Extraction Solvent Over Phenolic Profiles

Values presented in Table 2, Table 3 and Table 4 are divided according to the sources of variability (factors): drying temperature (DT) and extraction solvent (ES). Results for each factor level (different temperatures or different extraction solvents) were obtained as the mean of all levels of the second factor, which explains the high magnitude of the standard deviation. When studying two factors simultaneously, the significant interaction among each other is frequently observed. Therefore, the interaction (DT × ES) among the two factors studied herein was evaluated to verify if the differences in phenolic compound concentrations achieved with different solvents depended on the temperature to which the plant tissues were exposed throughout the drying process. As it might be concluded from Table 2, the interaction was always significant (*p* < 0.001), not allowing presentation of the statistical classification resulting from the effect of each individual factor. Nevertheless, some overall conclusions could be achieved after the analysis of the estimated marginal mean (EMM) plots obtained for each phenolic compound.

In the case of *C. urucurana* leaves (Table 2), which presented quercetin-3-*O*-rutinoside, apigenin-6-*C*-glucoside, and (epi)catechin dimer II as the major compounds, the effect of DT was less pronounced than that corresponding to ES. This conclusion is based not only in the fact that compounds **9**, **12**, **15**, **20,** and **21**, did not present significant differences pertaining DT, but also because most conclusions obtained from the EMM plots are linked to the effect of ES. 

In fact, it could be inferred that compounds **9**, **12**, **15**, **17**, **19**–**22** and total phenolics were obtained in higher concentrations (independently of DT) when the extraction was done with the hydroethanolic extracts. This tendency was also observed among compounds **5** and **7** (except at 60 °C). Despite the lack of similar results in the effects of DT, it is possible to indicate 50 °C as the most adequate temperature, considering the higher concentration in total phenolics obtained in this case.

The major phenolics in the barks of *C. urucurana* (Table 3), independent of DT or ES, were the (epichatechin) dimers (compounds **4** and **5**) and catechin (compound **6**). As observed in the experiments conducted on the leaves, the hydroethanolic mixture proved to be a better extracting solvent than water alone, as validated by the higher concentrations of compounds **4**–**6**, **9**, **17**, **18**, **20,** and **25** (and also the higher concentrations of total phenolics) obtained with the hydroalcoholic solvent. Once again, the effect of DT was less obvious, as no other unequivocal differences except the higher concentrations of compounds **2**, **5**, and **6** in bark dried at 40 °C could be observed in the EMM plots.

The stems of *C. urucurana* (Table 4) showed lower overall quantities of phenolic compounds, despite the significantly high levels of (epi)catechin dimer I, the main phenolic compound by far. The factors under study (DT and ES) were shown once again to exert their effect in a cooperative manner (significant interaction in all cases, except quercetin-*O*-hexosyl-rutinoside III). Nonetheless, it became evident that hydroalcoholic extracts (independent of DT) allowed higher yields of all identified phenolic compounds (except gallic acid), which was in agreement with the results obtained with the leaves and bark. In DT, the effects were not equally obvious, except for the general tendency for lower extraction yields in the case of samples dried at 70 °C and higher yields among those dried at 103 °C.

### 2.3. Linear Discriminant Analysis

All previous conclusions were obtained considering the individual effects of the studied factors over each individual botanical part. To complement the study, it was also interesting to evaluate the effects of both factors over phenolic profiles independently of being extracted from leaves, bark, or leaves. To achieve this purpose, the linear discriminant analysis (LDA) was employed. From a mathematical point of view, LDA evaluated the correlations among the studied factors (DT and ES), which represent the categorical dependent variables, and the concentrations of all quantified phenolic compounds, herein considered as the quantitative independent variables. One of the contributions of LDA is selecting which of these independent variables showed the most significant changes, a process that might be achieved through Wilks’ λ test. The classification performance of each variable was considered significant for *p-*values below 0.050.

**Figure 1:**
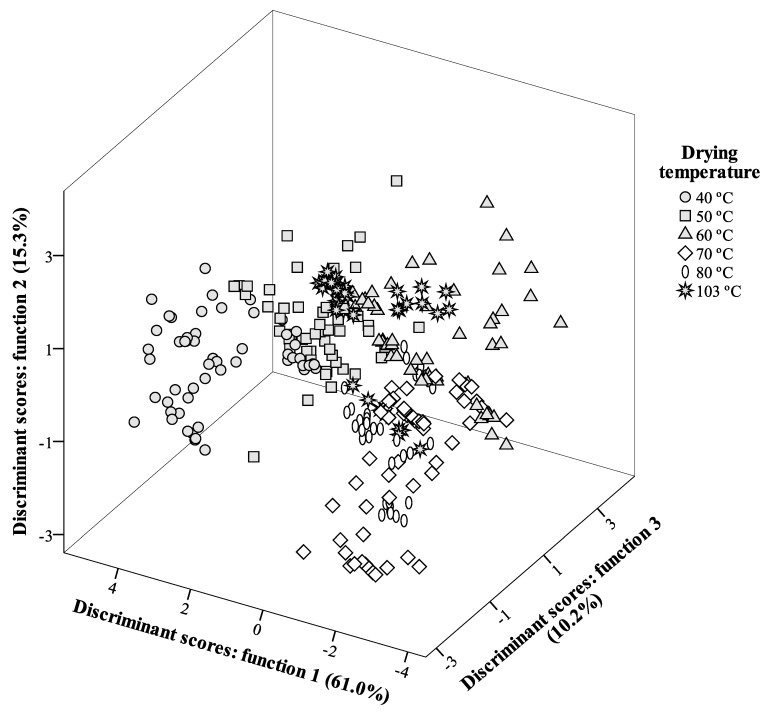
Spatial distribution of DT (drying temperature) and ES (extraction solvent) markers following the distribution set by the discriminant functions coefficients. Function 1 accounted for 61.0% of the variation, function 2 accounted for 15.3%, while function 3 accounted for 10.2%.

In the case of DT effects, the number of defined discriminant functions was five, owing to the fact that there were six levels for this factor (40 °C, 50 °C, 60 °C, 70 °C, 80 °C, 103 °C). The first three functions were plotted in Figure 1, and included 86.5% (first function: 61.0%; second function: 15.3%; third function: 10.2%) of the observed variance of data.

Among the 25 analyzed variables, the discriminant model excluded only quercetin-*O*-pentosyl-rutinoside (compound **12**), quercetin-*O*-hexosyl-rutinoside I (compound **13**), quercetin-*O*-hexosyl-rutinoside III (compound **16**), quercetin-*O*-deoxy-hexosyl-hexoside (compound **17**), quercetin-3-*O*-glucoside (compound **20**), and isorhamnetin-*O*-deoxy-hexosyl-hexoside (compound **24**) as having no discriminant effects.

Considering the 19 variables selected as contributors to discriminate different drying temperatures, (epi)gallocatechin showed the highest correlation with function 1 (positive side of function 1 axis in Figure 1), justifying the separation of markers corresponding to 40 °C, the temperature that allowed the highest contents (60 mg/100 g dw) of that phenolic compound.

Function 2, in turn, was mostly correlated with (epi)catechin dimer I, particularly noticeable in *C. urucurana* stems, in which this compound was obtained in much higher concentrations in samples dried at 103 °C (183 mg/100 g dw), especially when compared to the results obtained with samples dried at 70 °C (13 mg/100 g dw), and both myricetin derivatives (higher in samples dried at 103 °C and lower in those dried at 70 °C). Together, these differences were important to place markers corresponding to 103 °C in the positive side of the function 2 axis, while those belonging to 70 °C were located in the negative side (indicating the lower concentrations of the phenolic compounds were more correlated with this function).

In addition, function 3 was more strongly correlated to apigenin-6,8-*C*-di-hexoside, which was detected in higher concentrations in samples dried at 60 °C (positive end of function 3 axis in Figure 1).

A second LDA was performed, using ES as the statistical factor. Owing to the fact that this factor comprised only 2 levels, it was not possible to obtain any kind of plot, since at least two discriminant functions would be necessary, and the number of functions is equal to the number of levels minus 1. Nevertheless, it was possible to verify which variables exhibited discriminant abilities; those variables ordered according to their correlation (highest to lowest) with the single defined function were: (epi)catechin dimer II, isorhametin-3-*O*-rutinoside, catechin, quercetin-*O*-hexosyl-rutinoside I, isorhamnetin-*O*-deoxy-hexosyl-hexoside, quercetin-*O*-di-hexoside, (epi)gallocatechin–(epi)catechin, apigenin-6,8-*C*-di-hexoside, (epi)catechin tetramer, quercetin-*O*-deoxy-hexosyl-hexoside, apigenin-8-*C*-glucoside, myricetin-3-*O*-rutinoside, gallic acid, and quercetin-3-*O*-rutinoside. All the previous phenolic compounds (except (epi)gallocatechin–(epi)catechin) were obtained in higher concentrations when the *C. urucurana* samples were extracted with the hydroalcoholic mixture.

In this second LDA, the classification performance was 100% accurate, either for original grouped cases, as well as for the cross-validated grouped ones, which represent a strong indicator of the significant differences resulting from using different extraction solvents.

## 3. Materials and Methods

### 3.1. Standards and Reagents

HPLC grade acetonitrile (99.9%) was purchased from Fisher Scientific (Lisbon, Portugal). Formic acid was obtained from Sigma–Aldrich (St. Louis, MO, USA). Phenolic standards were acquired from Extrasynthèse (Genay, France). All other general laboratory reagents were purchased from Panreac Química S.L.U. (Barcelona, Spain). Water was treated in a Milli-Q water purification system (TGI Pure Water Systems, Sunnyvale, TX, USA).

### 3.2. Plant Material and Kinetic Temperature

The leaves, branches and bark of *C. urucurana*, collected from 7:30 a.m. to 8 a.m. in January 2017 from the rural area of Santo Antônio da Barra, Goiás, Brazil, were used as raw material. The samples were registered in the Herbarium of the Goiano Federal Institute–Rio Verde Campus, under number 602.

The drying process was carried out at the Post-Harvest Laboratory of Vegetable Products of the Goiano Federal Institute of Education, Science and Technology–Rio Verde Campus, Goiás, Brazil, with defoliation and selection operations. The drying was conducted in a forced air circulation oven (Marconi MA35, Marconi Equipment for Laboratory Ltd., Algodoal, Piracicaba - SP, Brazil), at different drying air temperatures: 40, 50, 60, 70, 80, and 130 °C (the last two temperatures, 80 and 130 °C, were only used for the branches and bark samples). All samples were comminuted in multiprocessor, reduced to a fine dry powder, and mixed to obtain homogenized samples.

### 3.3. Hydroethanolic Extract and Decoction Preparation

To prepare the hydroethanolic extracts, 1 g of each sample was submitted to extraction with an ethanol/water mixture (80:20, *v*/*v*; 30 mL) at 25 °C and 150 rpm during 1 h, followed by filtration through Whatman filter paper No. 4. Afterwards, the residue was extracted with one additional portion of the hydroethanolic mixture and the combined extracts were evaporated under reduced pressure (rotary evaporator Büchi R-210, Flawil, Switzerland).

For decoction preparation, the lyophilized plant material (500 mg) was added to 100 mL of distilled water, heated, and boiled for 5 min. The mixture was left to stand for 5 min and then filtered under reduced pressure.

The obtained hydroethanolic extracts and decoctions were lyophilized for further analysis of phenolic compounds.

### 3.4. Phenolic Composition of the Hydroethanolic Extracts and Decoctions

The phenolic profile was determined by HPLC–DAD–ESI/MSn (High-performance liquid chromatography coupled to electrospray ionization mass spectrometric detection in negative ion mode) (Dionex Ultimate 3000 UPLC, Thermo Scientific, San Jose, CA, USA). The phenolic compounds were separated and identified as previously described by Bessada et al. [19]. The obtained extracts were re-dissolved at a concentration of 5 mg/mL with an ethanol/water (80:20, *v*/*v*) mixture. A double online detection was performed using diode array detector (DAD) by selecting 280, 330, and 370 nm as the preferred wavelengths, and a mass spectrometer (MS) connected to the HPLC system via the DAD cell outlet. The MS detection was performed in negative mode, using a Linear Ion Trap LTQ XL mass spectrometer (ThermoFinnigan, San Jose, CA, USA) equipped with an ESI source.

The identification of phenolic compounds was performed based on their chromatographic behavior and UV-vis and mass spectra by comparison with standard compounds (when available) and data reported in the literature. Data acquisition was carried out with the Xcalibur^®^ data system (ThermoFinnigan, San Jose, CA, USA). For quantitative analysis, a calibration curve for each available phenolic standard was constructed based on the UV signal. The phenolic compounds for which a commercial standard was not available were quantified using calibration curves of the most similar standard compound: apigenin-6-*C*-glucoside (y = 107,025x + 61,531, R2 = 0.998); catechin (y = 84,950x − 23,200, R2 = 0.999); protocatechuic acid (y = 214,168x + 27,102, R2 = 0.999); quercetin-3-O-glucoside (y = 34843x − 160,173, R2 = 0.999); quercetin-3-O-rutinoside (y = 13343x + 76,751, R2 = 0.999). The results were expressed as mg/100 g of plant tissue dry weight (dw).

### 3.5. Statistical Analysis

Three samples were used for each group and all the assays were carried out in triplicate. The results were expressed as mean values ± standard deviation (SD).

Data were expressed as mean ± standard deviation, maintaining the significant numbers allowed by the magnitude of the corresponding standard deviation. An analysis of variance (ANOVA) with type III sums of squares, using the general linear model (GLM) procedure to compare differences in the concentrations of phenolic compounds, was employed. The 2-way ANOVA factors were “extraction solvent” (ES) and “drying temperature” (DT). When a statistically significant interaction among the two factors was verified, their effect was evaluated by from the estimated marginal means plots (considering all levels of each factor). Cases with no significant interaction were compared using Tukey’s multiple comparison test, after verifying the homogeneity of variances through Levene’s test. Differences among results obtained with each ES were compared through Student’s t-test, since less than three levels of this factor were available.

These analyses were carried out using IBM SPSS Statistics for Windows, Version 23.0. (IBM Corp., Armonk, New York, USA).

Complementarily, a linear discriminant analysis (LDA) was applied to evaluate the overall effects of ES and DT, independent of the *C. urucurana* tissue (leaf, bark, or stem) under study. The stepwise technique, considering Wilks’ **λ** test with the usual probabilities of *F* (3.84 to enter and 2.71 to be removed) for variable selection, was employed. With this procedure it was aimed to estimate the association between the single categorical dependent variables (ES or DT) and the quantitative independent variables (phenolic compound concentrations). Finding the independent variables with highest contribution to discriminate the average score profiles of different ES or DT allowed selection of the specific conditions that were expected to maximize the concentration of targeted phenolic compounds. In both cases, a leaving-one-out cross validation procedure was carried out to assess the model performance.

## 4. Conclusions

Besides the conclusions highlighting the higher efficiency of the hydroethanolic mixture instead of water alone, it was also possible to verify that the drying temperature allowed maximization of the yield in specific phenolic compounds. Specifically: (epi)gallocatechin was obtained in samples dried at 40 °C (the temperature that also allowed higher yields in total phenolic compounds in *C. urucurana* barks); (epi)catechin dimer I and myricetin derivatives were maximized in samples dried at 103 °C (which was also the temperature allowing higher yield in total phenolic compounds in *C. urucurana* stems); apigenin-6,8-*C*-di-hexoside, in turn, was obtained in higher concentrations in samples dried at 60 °C. In general, the effect of ES was more significant than that observed assaying different DTs. However, in addition to the identification of the best extraction solvent, it was also possible to detect the drying temperature that was expected to maximize the extraction yield of specific individual phenolic compounds. The maximization of the recovery of high-added value compounds from plant bio-residues (leaves, branches, and barks of *C. urucurana*) fulfils the necessary requirements for the development of a sustainable and profitable methodology for obtaining those same compounds, which can be further applied in various products and in different industrial segments, such as in the food, pharmaceutical, textile, and the agricultural industry, among others.

## Figures and Tables

**Table 1:** Retention time (Rt), wavelengths of maximum absorption in the visible region (λ_max_), mass spectral data, and tentative identification of the phenolic compounds present in *C. urucurana* Baill. leaves (L), branches (B), and bark (S).

Peak	Rt (min)	λ_max_ (nm)	Molecular Ion [M − H]^−^ (*m*/*z*)	MS^2^ (*m*/*z*)	Tentative Identification	Sample
1	4.40	270	169	125 (100)	Gallic acid	S
2	4.60	276	611	305 (100), 287 (11), 261 (41), 247 (14), 221 (87), 179 (50)	(Epi)gallocatechin ^A^	B
3	4.85	276	593	575 (5), 467 (12), 441 (8), 425 (100), 305 (2), 287 (8)	(Epi)gallocatechin–(epi)catechin ^A^	B
4	5.22	278	577	559 (10), 451 (23), 425 (100), 407 (22), 289 (11)	(Epi)catechin dimer I ^A^	B + L + S
5	5.99	279	577	559 (6), 451 (19), 425 (2), 407 (19), 289 (8)	(Epi)catechin dimer II ^A^	B + L + S
6	6.77	280	289	245 (100), 231 (9), 205 (36), 179 (13)	Catechin ^A^	B + L + S
7	7.12	279	1153	577 (55), 559 (15), 451 (5), 425 (5), 407 (4), 289 (6)	(Epi)catechin tetramer ^A^	L
8	13.05	331	593	473 (100), 431 (31), 353 (29), 341 (4), 311 (2)	Apigenin-6,8-*C*-di-hexoside ^B^	L
9	13.59	347	625	301 (100)	Quercetin-*O*-di-hexoside ^C^	B + L + S
10	14.18	352	625	317 (100)	Myricetin-*O*-deoxyhexosyl-hexoside ^D^	B + S
11	14.41	350	625	317 (100)	Myricetin-3-*O*-rutinoside ^D^	S
12	15.1	354	741	609 (13), 301 (100)	Quercetin-*O*-pentosyl-rutinoside ^C^	L
13	15.23	341	771	609 (27), 301 (100)	Quercetin-*O*-hexosyl-rutinoside I ^C^	S
14	15.61	342	771	609 (22), 301 (100)	Quercetin-*O*-hexosyl-rutinoside II ^C^	S
15	15.86	336	431	413 (2), 341 (5), 311 (100), 283 (3)	Apigenin-8-*C*-glucoside ^B^	L
16	15.89	329	771	301 (100)	Quercetin-*O*-hexosyl-rutinoside III ^C^	S
17	16.94	350	609	301 (100)	Quercetin-*O*-deoxyhexosyl-hexoside ^C^	B + L + S
18	17.17	356	609	301 (100)	Quercetin-3-*O*-rutinoside ^C^	B + L + S
19	17.54	337	431	413 (6), 341 (27), 311 (100), 283 (3)	Apigenin-6-*C*-glucoside ^B^	L
20	18.3	354	463	301 (100)	Quercetin-3-*O*-glucoside ^C^	B + L
21	18.98	348	593	285 (100)	Kaempferol-*O*-deoxyhexosyl-hexoside ^C^	L
22	20.29	346	593	285 (100)	Kaempferol-3-*O*-rutinoside ^C^	L + S
23	20.79	334	623	315 (100)	Isorhametin-3-*O*-rutinoside ^C^	S
24	21.3	329	623	315 (100)	Isorhamnetin-*O*-deoxyhexosyl-hexoside ^C^	S
25	21.86	339	653	345 (100)	Syringetin-*O*-rutinoside ^C^	B

Standard calibration curves: A: catechin (*y* = 84,950*x* − 23,200, *R^2^* = 0.9999); B: apigenin-6-glucoside (*y* = 107,025*x* + 61531, *R^2^* = 0.9989); C: quercetin-3-*O*-glucoside (*y* = 34843*x* − 160,173, *R^2^* = 0.9998); D: quercetin-3-*O*-rutinoside (*y* = 13343*x* + 76,751, *R^2^* = 0.9998).

**Table 2:** Phenolic compound quantification (mg/g extract) in *C. urucurana* Baill. leaves submitted to different drying and extraction conditions.

Compound	Tentative Identification (Standard Used for Quantification)	Quantification (mg/100 g dw − Dry Weight)								
		Drying Temperature (DT)				*p*-Value (*n* = 18)	Extraction Solvent (ES)		*p*-Value (*n* = 36)	DT × ES
		40 °C	50 °C	60 °C	70 °C		Water	Hydroalcoholic		*p*-Value (*n* = 72)
**4**	(Epi)catechin dimer I	43 ± 5	51 ± 8	35 ± 13	41 ± 2	<0.001	42 ± 4	43 ± 13	0.879	<0.001
**5**	(Epi)catechin dimer II	79 ± 18	163 ± 101	90 ± 7	172 ± 68	<0.001	82 ± 20	170 ± 81	<0.001	<0.001
**6**	Catechin	36 ± 14	61 ± 34	40 ± 3	75 ± 25	<0.001	48 ± 31	58 ± 22	0.109	<0.001
**7**	(Epi)catechin tetramer	13 ± 3	28 ± 3	13 ± 6	21 ± 12	<0.001	14 ± 10	23 ± 6	<0.001	<0.001
**8**	Apigenin-6,8-*C*-dihexoside	1 ± 1	0.7 ± 0.2	4 ± 1	1.0 ± 0.1	<0.001	2 ± 2	1 ± 1	0.001	<0.001
**9**	Quercetin-*O*-dihexoside	25 ± 2	24 ± 4	24 ± 5	23 ± 4	0.657	21 ± 2	27 ± 1	<0.001	<0.001
**12**	Quercetin-*O*-pentosyl-rutinoside	26 ± 1	25 ± 3	25 ± 3	25 ± 3	0.274	23 ± 2	27 ± 1	<0.001	<0.001
**15**	Apigenin-8-*C*-glucoside	69 ± 10	67 ± 16	69 ± 5	68 ± 6	0.950	60 ± 5	77 ± 5	<0.001	<0.001
**17**	Quercetin-*O*-deoxyhexosyl-hexoside	55 ± 6	60 ± 6	49 ± 7	62 ± 4	<0.001	51 ± 7	62 ± 5	<0.001	<0.001
**18**	Quercetin-3-*O*-rutinoside	292 ± 33	364 ± 41	354 ± 20	331 ± 21	<0.001	333 ± 24	338 ± 52	0.596	<0.001
**19**	Apigenin-6-*C*-glucoside	142 ± 36	104 ± 17	112 ± 12	102 ± 11	<0.001	98 ± 9	132 ± 27	<0.001	<0.001
**20**	Quercetin-3-*O*-glucoside	33 ± 2	33 ± 5	32 ± 2	32 ± 3	0.877	30 ± 2	35 ± 2	<0.001	<0.001
**21**	Kaempferol-*O*-deoxyhexosyl-hexoside	30 ± 2	28 ± 3	28 ± 4	28 ± 4	0.410	26 ± 2	31 ± 1	<0.001	0.001
**22**	Kaempferol-3-*O*-rutinoside	53 ± 4	49 ± 5	48 ± 2	48 ± 5	0.001	47 ± 3	53 ± 3	<0.001	<0.001
	**Total phenols**	**897 ± 71**	**1059 ± 235**	**922 ± 18**	**1029 ± 110**	**0.001**	**877 ± 50**	**1077 ± 148**	**<0.001**	**<0.001**

**Table 3:** Phenolic compound quantification in *C. urucurana* Baill. bark submitted to different drying and extraction conditions.

Compound	Tentative Identification (Standard Used for Quantification)	Quantification (mg/100 g dw)										
		Drying Temperature (DT)						*p*-Value (*n* = 18)	Extraction Solvent (ES)		*p*-Value (*n* = 54)	DT × ES
		40 °C	50 °C	60 °C	70 °C	80 °C	103 °C		Water	Hydroalcoholic		*p*-Value (*n* = 108)
**2**	(Epi)gallocatechin	60 ± 13	30 ± 9	11 ± 7	18 ± 3	18 ± 2	5 ± 5 *	<0.001	21 ± 14	27 ± 24	0.105	<0.001
**3**	(Epi)gallocatechin–(epi)catechin	36 ± 8	12 ± 1	14 ± 4	20 ± 12	20 ± 19	11 ± 11 *	<0.001	25 ± 9	12 ± 15	<0.001	<0.001
**4**	(Epi)catechin dimer I	56 ± 17	34 ± 14	46 ± 8	55 ± 6	35 ± 13	30 ± 24	<0.001	48 ± 31	58 ± 22	<0.001	<0.001
**5**	(Epi)catechin dimer II	75 ± 28	24 ± 15	17 ± 9	23 ± 7	18 ± 11	9 ± 4	<0.001	16 ± 15	40 ± 29	<0.001	<0.001
**6**	Catechin	81 ± 46	36 ± 25	33 ± 9	42 ± 12	25 ± 10	12 ± 6	<0.001	21 ± 11	56 ± 34	<0.001	<0.001
**9**	Quercetin-*O*-dihexoside	18 ± 2	17 ± 2	19 ± 2	17 ± 3	16 ± 3	15 ± 6	0.006	14 ± 2	20 ± 1	<0.001	<0.001
**10**	Myricetin-*O*-deoxyhexosyl-hexoside	19 ± 2	17 ± 2	18 ± 2	17 ± 3	15 ± 2	9 ± 1*	<0.001	14 ± 3	16 ± 7	0.048	<0.001
**17**	Quercetin-*O*-deoxyhexosyl-hexoside	18 ± 2	17 ± 2	18 ± 2	17 ± 3	15 ± 2	14 ± 5	<0.001	14 ± 3	19 ± 1	<0.001	<0.001
**18**	Quercetin-3-*O*-rutinoside	19 ± 2	16 ± 2	19 ± 2	17 ± 3	15 ± 2	15 ± 5	<0.001	14 ± 3	19 ± 2	<0.001	<0.001
**20**	Quercetin-3-*O*-glucoside	18 ± 2	17 ± 2	18 ± 2	17 ± 3	15 ± 2	14 ± 5	<0.001	14 ± 2	19 ± 1	<0.001	<0.001
**25**	Syringetin-*O*-rutinoside	19 ± 2	16 ± 2	18 ± 2	17 ± 3	15 ± 2	14 ± 5	<0.001	14 ± 3	19 ± 1	<0.001	<0.001
	**Total phenolics**	**420 ± 125**	**235 ± 77**	**231 ± 39**	**260 ± 27**	**207 ± 30**	**144 ± 40**	**<0.001**	**195 ± 61**	**304 ± 115**	**<0.001**	**<0.001**

* This compound was not detected in ethanolic extracts at this temperature.

**Table 4:** Phenolic compound quantification in *C. urucurana* Baill. stems submitted to different drying and extraction conditions.

Compound	Tentative Identification (Standard Used for Quantification)	Quantification (mg/100 g dw)										
		Drying Temperature (DT)						*p*-Value (*n* = 18)	Extraction Solvent (ES)		*p*-Value (*n* = 54)	DT × ES
		40 °C	50 °C	60 °C	70 °C	80 °C	103 °C		Water	Hydroalcoholic		*p*-Value (*n* = 108)
**1**	Gallic acid	2 ± 1	2 ± 1	1 ± 1	nd	4 ± 1 *	0.5 ± 0.1	<0.001	1 ± 1	1 ± 1	0.184	<0.001
**4**	(Epi)catechin dimer I	107 ± 71	137 ± 34	125 ± 63	13 ± 1 *	50 ± 39	183 ± 62	<0.001	59 ± 43	143 ± 80	<0.001	<0.001
**5**	(Epi)catechin dimer II	22 ± 1	33 ± 1	2.2 ± 0.1	21 ± 1	18 ± 1	18 ± 1	0.003	nd	19 ± 9	-	-
**6**	Catechin	6 ± 5	6 ± 5	3 ± 2	1 ± 1	0.7 ± 0.2	5 ± 2	<0.001	1 ± 1	6 ± 4	<0.001	<0.001
**9**	Quercetin-*O*-dihexoside	1.0 ± 0.3	0.9 ± 0.2	0.7 ± 0.3	0.6 ± 0.3	0.6 ± 0.3	1.0 ± 0.2	<0.001	0.5 ± 0.2	1.1 ± 0.2	<0.001	<0.001
**10**	Myricetin-*O*-deoxyhexosyl-hexoside	1.1 ± 0.4	1.1 ± 0.3	0.8 ± 0.3	0.5 ± 0.3	0.6 ± 0.2	1.1 ± 0.2	<0.001	0.6 ± 0.2	1.1 ± 0.3	<0.001	<0.001
**11**	Myricetin-3-*O*-rutinoside	1.1 ± 0.4	1.0 ± 0.3	0.7 ± 0.3	0.5 ± 0.3	0.6 ± 0.3	1.0 ± 0.1	<0.001	0.5 ± 0.2	1.1 ± 0.2	<0.001	<0.001
**13**	Quercetin-*O*-hexosyl-rutinoside I	1.0 ± 0.3	1.0 ± 0.2	0.7 ± 0.3	0.5 ± 0.3	0.6 ± 0.2	1.0 ± 0.2	<0.001	0.5 ± 0.2	1.0 ± 0.2	<0.001	<0.001
**14**	Quercetin-*O*-hexosyl-rutinoside II	1.0 ± 0.3	0.9 ± 0.2	0.7 ± 0.3	0.5 ± 0.3	0.6 ± 0.3	1.0 ± 0.2	<0.001	0.6 ± 0.2	1.1 ± 0.2	<0.001	<0.001
**16**	Quercetin-*O*-hexosyl-rutinoside III	1.0 ± 0.3c	1.2 ± 0.3b	0.8 ± 0.3d	0.6 ± 0.3e	0.6 ± 0.3e	1.3 ± 0.3a	<0.001	0.6 ± 0.3	1.2 ± 0.3	<0.001	0.870
**17**	Quercetin-*O*-deoxyhexosyl-hexoside	2 ± 1	3 ± 1	2 ± 1	0.7 ± 0.4	0.8 ± 0.4	2.6 ± 0.5	<0.001	1 ± 1	3 ± 1	<0.001	<0.001
**18**	Quercetin-3-*O*-rutinoside	4 ± 2	6 ± 3	4 ± 2	1 ± 1	1 ± 1	6 ± 2	<0.001	2 ± 1	5 ± 2	<0.001	<0.001
**22**	Kaempferol-3-*O*-rutinoside	0.9 ± 0.3	0.9 ± 0.3	0.7 ± 0.3	1.2 ± 0.5	1.1 ± 0.5	1.6 ± 0.5	<0.001	0.6 ± 0.2	1.6 ± 0.4	<0.001	<0.001
**23**	Isorhamnetin-3-*O*-rutinoside	1.1 ± 0.5	1.2 ± 0.3	1.3 ± 0.5	4 ± 3	3 ± 2	3 ± 2	<0.001	1.0 ± 0.4	4 ± 2	<0.001	<0.001
**24**	Isorhamnetin-*O*-deoxyhexosyl-hexoside	0.9 ± 0.3	1.0 ± 0.3	0.8 ± 0.3	0.5 ± 0.2	0.6 ± 0.2	1.1 ± 0.2	<0.001	0.5 ± 0.2	1.1 ± 0.2	<0.001	<0.001
	**Total phenolics**	**142 ± 95**	**179 ± 62**	**144 ± 74**	**29 ± 12**	**72 ± 51**	**218 ± 79**	**<0.001**	**70 ± 47**	**191 ± 86**	**<0.001**	**<0.001**

* This compound was not detected in ethanolic extracts at this temperature.

## References

[1] Franco E.A.P., Barros R.F.M. (2006). Uso e diversidade de plantas medicinais no Quilombo Olho D’agua dos Pires, Esperantina, Piauí. Rev. Bras. Plantas Med..

[2] Vila Verde G.M., Paula J.R., Caneiro D.M. (2003). Levantamento etnobotânico das plantas medicinais do cerrado utilizadas pela população de Mossâmedes (GO). Rev. Bras. Farmacogn..

[3] Maciel M.A.M., Pinto A.C., Veiga V.F., Grynberg N.F., Echevarria A. (2002). Plantas medicinais: A necessidade de estudos multidisciplinares. Quim. Nova.

[4] Souza C.D. De, Felfili J.M. (2006). Uso de plantas medicinais na região de Alto Paraíso de Goiás, GO, Brasil. Acta Botânica Bras..

[5] Soldera C.C., Zanella G.N., Frasson A.P.Z. (2013). Avaliação da atividade antibacteriana de *Croton urucurana*. Rev. Context. Saúde.

[6] Dos Santos Carvalho G., Da Silva L.S., Silva L.B., Dos Santos Almeida M.L., Pavam B.E., Peres M.T.L.P. (2014). Mortalidade e comprometimento do desenvolvimento de *Zabrotes subfasciatus* Boh. (Coleoptera: Chrysomelidae), induzido pelo extrato de sangra d’água *Croton urucurana* Baill (Euphorbiaceae). Comun. Sci..

[7] Rao V.S., Gurgel L.A., Lima-Júnior R.C.P., Martins D.T.O., Cechinel-Filho V., Santos F.A. (2007). Dragon’s blood from *Croton urucurana* (Baill.) attenuates visceral nociception in mice. J. Ethnopharmacol..

[8] Koche D., Shirsat R., Imran S., Bhadange D.G. (2010). Phytochemical screening of eight traditionally used ethnomedicinal plants from Akola district (MS) India. Int. J. Pharma Bio Sci..

[9] Pizzolatti M.G., Bortoluzzi A.J., Brighente I.M.C., Zuchinalli A., Carvalho F.K., Candido A.C.S., Peres M.T.L.P. (2013). Clerodane diterpenes from bark of *Croton urucurana* baillon. J. Braz. Chem. Soc..

[10] Goneli A.L.D., Pire O.V., Vilhasanti H.D.C.B., Gonçalves A.A. (2014). Modelagem matemática e difusividade efetiva de folhas de aroeira durante a secagem. Pesqui. Agropecu. Trop..

[11] Tabaldi L.A., Vieira M.D.C., Zárate N.A.H., Silva L.R.D., Gonçalves W.L.F., Pilecco M., Formagio A.S.N., Gassi R.P., Padovan M.P. (2012). Cover crops and their effects on the biomass yield of *Serjania marginata* plants. Ciência Rural.

[12] Sousa F.C., Martins J.J.A., Rocha A.P.T., Gomes J.P., Pessoa T., Martins J.N. (2015). Predição de modelos sobre a cinética de secagem de folhas de Ziziphus joazeiro Mart. Rev. Bras. Plantas Med..

[13] Santos-Buelga C., Gonzalez-Manzano S., Dueñas M., Gonzalez-Paramas A.M., Sarker S., Nahar L. (2012). Extraction and isolation of phenolic compounds. Natural Products Isolation. Methods in Molecular Biology (Methods and Protocols)—Chapter 17.

[14] Furlan C.M., Santos K.P., Sedano-Partida M.D., Motta L.B. da, Santos D.Y.A.C., Salatino M.L.F., Negri G., Berry P.E., van Ee B.W., Salatino A. (2015). Flavonoids and antioxidant potential of nine Argentinian species of Croton (Euphorbiaceae). Rev. Bras. Bot..

[15] Cordeiro K.W., Felipe J.L., Malange K.F., Do Prado P.R., De Oliveira Figueiredo P., Garcez F.R., De Cássia Freitas K., Garcez W.S., Toffoli-Kadri M.C. (2016). Anti-inflammatory and antinociceptive activities of Croton urucurana Baillon bark. J. Ethnopharmacol..

[16] Ferreres F., Silva B.M., Andrade P.B., Seabra R.M., Ferreira M.A. (2003). Approach to the study of C-glycosyl flavones by ion trap HPLC-PAD-ESI/MS/MS: Application to seeds of quince (*Cydonia oblonga*). Phytochem. Anal..

[17] Dias M.I., Barros L., Fernandes I.P., Ruphuy G., Oliveira M.B.P., Santos-Buelga C., Barreiro M.F., Ferreira I.C.F.R. (2015). A bioactive formulation based on Fragaria vesca L. vegetative parts: Chemical characterisation and application in κ-carrageenan gelatin. J. Funct. Foods.

[18] Escobar-Avello D., Lozano-Castellón J., Mardones C., Pérez A.J., Saéz V., Riquelme S., Von Baer D., Vallverdú-Queralt A. (2019). Phenolic profile of grape canes: Novel compounds identified by LC-ESI-LTQ-orbitrap-MS. Molecules.

[19] Bessada S.M.F., Barreira J.C.M., Barros L., Ferreira I.C.F.R., Oliveira M.B.P.P. (2016). Phenolic profile and antioxidant activity of Coleostephus myconis (L.) Rchb.f.: An underexploited and highly disseminated species. Ind. Crops Prod..

